# Microvascular Ultrasonic Imaging of Angiogenesis Identifies Tumors in a Murine Spontaneous Breast Cancer Model

**DOI:** 10.1155/2020/7862089

**Published:** 2020-02-06

**Authors:** Sarah E. Shelton, Jodi Stone, Fei Gao, Donglin Zeng, Paul A. Dayton

**Affiliations:** ^1^Joint Department of Biomedical Engineering, UNC Chapel Hill and NC State Raleigh, 27599, USA; ^2^Department of Biostatistics, UNC Chapel Hill, 27599, USA

## Abstract

The purpose of this study is to determine if microvascular tortuosity can be used as an imaging biomarker for the presence of tumor-associated angiogenesis and if imaging this biomarker can be used as a specific and sensitive method of locating solid tumors. Acoustic angiography, an ultrasound-based microvascular imaging technology, was used to visualize angiogenesis development of a spontaneous mouse model of breast cancer (*n* = 48). A reader study was used to assess visual discrimination between image types, and quantitative methods utilized metrics of tortuosity and spatial clustering for tumor detection. The reader study resulted in an area under the curve of 0.8, while the clustering approach resulted in the best classification with an area under the curve of 0.95. Both the qualitative and quantitative methods produced a correlation between sensitivity and tumor diameter. Imaging of vascular geometry with acoustic angiography provides a robust method for discriminating between tumor and healthy tissue in a mouse model of breast cancer. Multiple methods of analysis have been presented for a wide range of tumor sizes. Application of these techniques to clinical imaging could improve breast cancer diagnosis, as well as improve specificity in assessing cancer in other tissues. The clustering approach may be beneficial for other types of morphological analysis beyond vascular ultrasound images.

## 1. Introduction

Clinical detection and diagnosis of breast cancer typically begins with X-ray mammographic identification of a suspicious lesion, followed by diagnostic imaging with other imaging modalities, often ultrasound. Mammography and grayscale ultrasound utilize anatomic features of breast tissue in order to discriminate malignant from benign breast tissue. The Breast Imaging-Reporting and Data System (BI-RADS) provides a framework for lesion ranking by probability of disease based on a number of radiographic features [1, 2]. Although reported numbers are variable, the sensitivity of screening mammography typically lies between 70% and 90% [3]. Ultrasound imaging can improve this sensitivity, although the specificity of breast imaging using this combination is poor in women with dense breasts, as low as 40% [3]. Furthermore, this limitation is even more significant with women with prosthetic implants, where X-ray mammography cannot penetrate silicone or saline effectively. Universally, uncertain imaging results require biopsy, which provides additional emotional stress, cost, and possibility of complication to a large percentage of women who end up with negative biopsy results after inconclusive imaging [4, 5]. Additional imaging methods that could better identify malignancies could improve breast cancer detection and ultimately reduce the number of unnecessary biopsies performed each year.

Microvascular imaging approaches have been proposed as an approach for cancer detection and diagnosis due to the prevalence of pathological angiogenesis, a process in which growing tumors quickly outpace their blood supply and trigger the growth of new vasculature [6]. Angiogenesis is recognized as a hallmark of cancer and a pervasive feature of solid tumors. Notably, the newly formed vessels are distinct from healthy vasculature in several ways [7]. Cancer neovasculature lacks regular hierarchical branching structures, leading to unpredictable flow and frequent arteriovenous shunts. The vessels are also immature and leaky and form tortuous, sinuous, and erratically shaped vessels [8–10]. Thus, perfusion patterns and abnormal vascular geometry make likely biomarkers for improving sensitivity to disease.

Acoustic angiography is a method of contrast-enhanced ultrasound imaging, and it detects broadband superharmonic emissions from microbubbles by transmitting at a low frequency and receiving with a higher frequency transducer, several harmonics above that of the transmitted signal [11]. Tissue produces much less energy than microbubbles in the superharmonic range, so imaging microbubbles with superharmonics enables high contrast-to-tissue ratios and high resolution due to the high frequency receive [12, 13]. The resulting images reveal that the morphology of vessels resolved at 100-200 *μ*m in diameter and contrast signal from vasculature with very little background signal. Acoustic angiography has been utilized in small animal models where it has enabled visualization of cancer-induced microvascular angiogenesis and invasion. Using this technique, researchers have observed microvascular patterns which exhibit significant differences in both morphology and density of microvasculature, in both rat and mouse tumor models [14–21]. It was also recently demonstrated to be feasible for imaging microvasculature in humans, depicting breast vasculature as small as 200 *μ*m to a depth of approximately 15 mm [22].

Other ultrasound imaging approaches have been used for visualizing tumor vasculature in clinical applications. Previous studies with Doppler and contrast-Doppler images of breast lesions have found that the detection of penetrating vessels is highly suggestive of malignancy [23–25]. Other features visible in Doppler imaging that suggested malignancy were hypervascularity and tortuosity. However, these studies lacked specificity and several false positives occurred (primarily in cases of fibroadenomas). Contrast-enhanced ultrasound imaging has also been investigated for breast cancer diagnosis, and features of contrast-enhanced ultrasound (CEUS) imaging have been found to be correlated with histological tumor features, especially microvessel density [26]. Additionally, reader studies investigating clinical CEUS imaging in tumors of other organs have shown that CEUS is superior to unenhanced imaging in the liver [27–31], pancreas [32, 33], kidney [34], and bladder [35]. Though the majority of ultrasound contrast studies are performed in 2D, Luo et al. acquired 3D CEUS images of liver lesions and processed them to create “sonographic angiograms” to display vascular structure [36]. They reported good reader agreement, sensitivity, and specificity using diagnostic features including ring-like enhancement, peripheral nodular enhancement, and spoke-and-wheel arterial arrangements, combined with the presence of early-phase enhancement.

Acoustic angiography can display 3D vascular morphology of vessels as small as 100-200 *μ*m in diameter with high contrast and provide information about vascular density for vessels of all sizes. Therefore, we hypothesize that acoustic angiography images may provide a sensitive and specific diagnostic tool for cancer through the evaluation of vascular features that are common in tumors.

This hypothesis has been tested here using two detection approaches in a genetically engineered mouse model of breast cancer. First, visual assessment with a reader study mimics clinical practices for image-based diagnosis. Second, a quantitative method uses spatial and geometric information (tortuosity) to detect tumor presence using a statistical clustering algorithm. The sensitivity and specificity of each method are reported using receiver operating characteristic (ROC) curves, and the relationships between robust lesion identification and tumor size are explored.

## 2. Materials and Methods

The C3(1)/Tag mouse is a model of basal breast cancer in which tumors develop spontaneously in the mammary pads of female mice, becoming palpable around 16 weeks of age [37]. Age-matched control mice were female wild-type (FVB/NJ) littermates. This study included 48 mice: 31 C3(1)/Tag (tumors) and 17 FVB/NJ (controls). Mice were monitored for tumor development using palpation and B-mode imaging, and contrast images were acquired in mice between 12 and 18 weeks old, as tumors emerged at different times. The Institutional Animal Care and Use Committee approved all protocols to ensure ethical standards for the treatment of animals and compliance with federal regulations.

### 2.1. Image Acquisition

Images of the mammary pads in the lower abdomen were collected while mice were anesthetized with 1.5% vaporized isoflurane in oxygen, and temperature was maintained with a heated stage. A 27-gauge catheter was inserted into a tail vein for the administration of contrast using a syringe pump (5 × 10^8^ microbubbles per minute, Harvard Apparatus, PHD2000). Fur was removed using clippers and chemical depilation, and ultrasound gel was applied to the imaging region.

Images were acquired with a modified Vevo 770 (VisualSonics, Toronto, ON, CA) using a custom dual-element RMV 707 which included a confocal 4 MHz annular element transducer for transmission in addition to a 30 MHz receive element. The driving waveform was a single-cycle, 1.2 MPa, 4 MHz pulse. Microbubbles used for this study were lipid-shelled, perfluorocarbon agents similar to Definity (Lantheus Medical Imaging 2017), approximately 1 *μ*m in diameter, made in-house as described previously [16].

Contrast images resulted from transmitting with the low frequency element and receiving the superharmonic signals from excited microbubbles with the high frequency element, and three-dimensional images were acquired using a linear translation stage. Two images were averaged at each location, with 100 *μ*m separating each frame. The frame rate was 4 frames per second, which resulted in an acquisition time of approximately 1-2 minutes for the 3D images encompassing 25 *mm* × 25 *mm* in the axial and lateral directions and 25-30 mm in the elevation direction. Tumor dimensions were measured in 3 orthogonal axes from the B-mode images. Tumor volumes were computed assuming ellipsoidal form, and the geometric mean was used to represent the average tumor diameter.

### 2.2. Qualitative Assessments

Visual image classification was performed using a reader study design, and seven readers with experience visualizing ultrasound images rated the likelihood of malignancy on a scale of 1-6. A user interface was created using GUIDE (Graphical User Interface Development Environment, MATLAB, The MathWorks, Inc.) to display 3 views of each contrast image, which were presented to readers in random order. Two views were 2D images in the axial and coronal planes with scrollbars to allow for interactive viewing of individual slices from the 3D image volume. The third view was a maximum intensity projection of the data in the coronal plane. Each image also displayed a small red dot, indicating the suspicious region for the readers to evaluate. In tumor images, the dot was placed at the center of the tumor. In control images, the dot was positioned randomly in the mammary pad. Localization was performed on the B-mode images prior to study initiation, and coordinates were transferred to the coregistered angiography images.

Readers were presented with the acoustic angiography contrast images in random order and did not have access to B-mode images. Readers used patterns of high vascular density, peripheral enhancement, tortuosity, tissue distortion, and branching as indicators of tumor presence. The presence of multiple features or very strong features increased the readers' confidence of malignancy.

Statistical analysis included calculation of receiver operating characteristic curves and interreader agreement and was performed using STATA/SE 14.2, treating reader assessments as ordinal variables. To characterize the relationship between the readers' performance and tumor diameter, we applied ROC regression. The general ROC regression model can be written as

(1) $$\begin{array}{c} {\text{ROC}}_{z}\left(p\right)=\Phi \left\{\alpha_{1}+\alpha_{2}{\bar{F}}_{0,x}^{-1}\left(p\right)+\beta^{\prime }z\right\}, \end{array}$$

where$\;{\bar{F}}_{0,x}\;$is the survival function for controls with covariate *x*, *z* = (*x*, *x*_*D*_), and *x* and *x*_*D*_ are common and disease-specific covariates, respectively. In the present study, we considered the ROC regression model

(2) $$\begin{array}{c} {ROC}_{z}\left(p\right)=\Phi \left\{\overset{K}{\underset{k=1}{\sum }}R_{k}\alpha_{0k}+\alpha_{1}\right.\overset{K}{\underset{k=1}{\sum }}R_{k}\hat{{\bar{F}}_{0,k}^{-1}}\left(p\right)+\left.\beta x\right\}, \end{array}$$

where $\hat{{\bar{F}}_{0,k}^{-1}}$ is the empirical survival function for the controls estimated for each reader *k*. The regression model was performed using the rocreg fuction, and standard errors were obtained using bootstrapping with 1000 replicates.

### 2.3. Quantitative Tortuosity Analysis

The images were also analyzed using quantitative methods to assess vascular tortuosity. First, contrast images were exported and linearly interpolated in the elevation direction to create isotropic voxels. Then, vessels were segmented using semiautomated multiscale ridge regression, using Aylward and Bullitt's algorithm [38]. Finally, tortuosity was calculated from the centerline of each vessel using two metrics known as the distance metric and the sum of angles metric after resampling the centerlines to 50 *μ*m spacing between points [39].

The distance metric (DM) is defined as the cumulative distance between consecutive points, *p*, along the centerline, divided by the distance between the endpoints:

(3) $$\begin{array}{c} \text{DM}=\frac{\sum_{x=1}^{n-1}\left\|p_{x}-p_{x+1}\right\|}{\left\|p_{x}-p_{n}\right\|}. \end{array}$$

The sum of angles metric (SOAM) is the sum of the angles between consecutive trios of points along the centerline, normalized by the path length. The angle is calculated by using the dot product of the two vectors defined by three points, normalized to unit length, then calculating the angle in radians using inverse cosine:

(4) $$\begin{array}{c} SOAM=\frac{\sum_{x=1}^{n-2}\cos^{-1}\left(\nu_{x}/\left\|\nu_{x}\right\|\cdot \nu_{x+1}/\left\|\nu_{x+1}\right\|\right)}{\sum_{x=1}^{n-1}\left\|p_{x}-p_{x+1}\right\|} \end{array}$$

Summary statistics of tortuosity parameters and ROC curves were calculated in R using the pROC and ROCR packages [40–42]. Clustering analysis was performed using the DBSCAN algorithm using the DBSCAN package in R [43], with a minimum number of points per cluster of 4 and a neighborhood size (*ϵ*) of 27.

## 3. Results and Discussion

**Figure 1:**
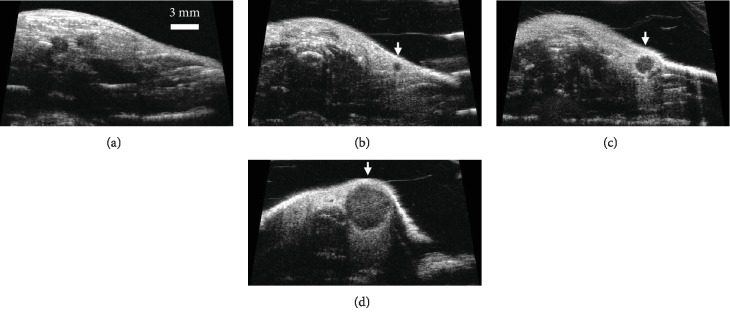
Frames of high frequency B-mode images acquired in the abdomen of (a) control FVB/N and tumor-bearing (b–d) C3(1)\Tag mice. The tumors shown in (b–d) are 0.8, 2.1, and 5.1 mm in diameter, respectively.

The tumors included in this study ranged from 0.8 mm to 8.2 mm in diameter with a mean and standard deviation of 3.1 ± 2.20 mm. The median size was 2.3 mm, and the size distribution was skewed toward small tumors (<3 mm) in order to assess the efficacy of ultrasound vascular imaging for tumor identification and to determine the influence of tumor size on sensitivity. Tumors smaller than 3 mm in diameter are barely palpable in the mammary pads of mice but visible in high frequency (30 MHz) B-mode imaging, as shown in Figure 1, which illustrates a typical image in the abdomen of a healthy control as well as images from mice with tumors 0.8, 2.1, and 5.1 mm in diameter.

**Figure 2:**
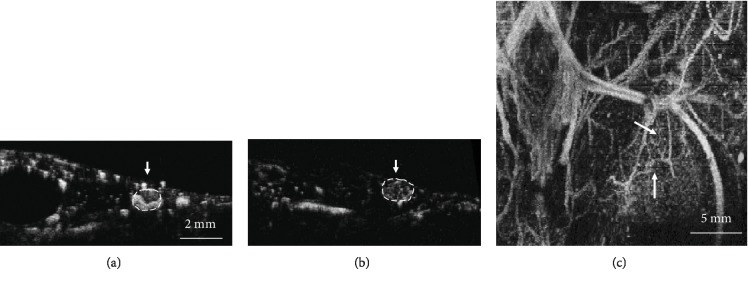
Typical contrast images of microtumors. (a, b) 1.5 and 1.7 mm tumors displaying patterns of vascular density, tortuosity, and peripheral enhancement in axial frames. (c) Maximum intensity projection in the coronal view from (b).

**Figure 3:**
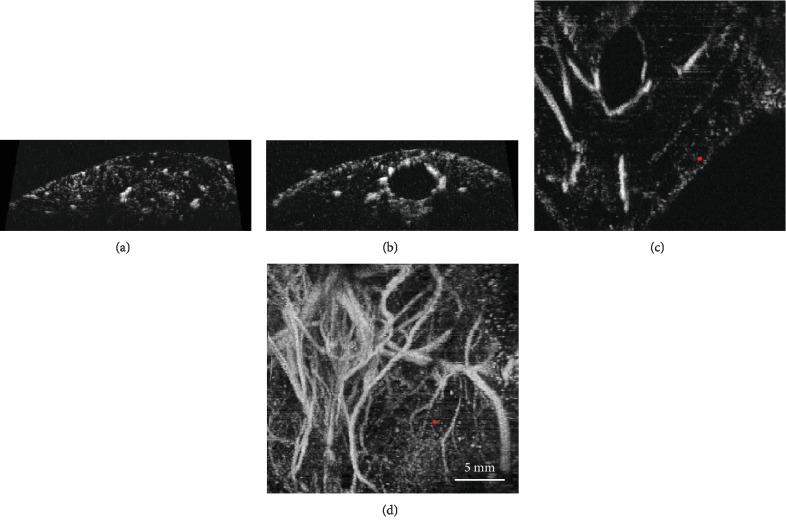
(a, b) Axial frames from control mice. (c) Coronal frame with a red dot to guide the reader's focus for assessment. (d) is the maximum intensity projection image corresponding to (c).

Acoustic angiography contrast images, such as those shown in Figures 2 and 3, reveal vascular cross-sections in the axial and coronal planes and show longer vessel segments in coronal planes and maximum intensity projections.  Figure 2 illustrates typical contrast images of small tumors less than 2 mm in diameter. Figures 2(a) and 2(b) show axial cross-sections through each tumor, with dense vasculature and peripheral enhancement apparent.  Figure 2(c) shows a coronal projection, showing vascular tortuosity and branching. These features of vascular geometry are not seen in control images, such as those shown in Figure 3.

### 3.1. Reader Study Analysis

Readers independently completed their assessments for all images in approximately two hours. There was strong agreement between the seven readers, reflected by a value of 0.778 for Kendall's coefficient of concordance, and no significant difference was found between reader effects.

**Figure 4:**
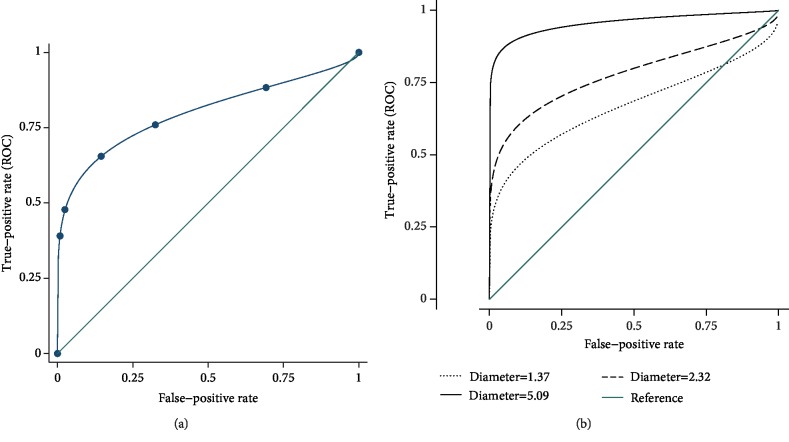
(a) Overall ROC curve derived from the combined assessment of all 7 readers (AUC = 0.80). (b) Expected ROC curves for 3 different tumor sizes (AUC = 0.67, 0.78, and 0.96).

A receiver operator characteristic curve was constructed from the aggregate results of all seven readers, which revealed an overall area under the curve of 0.798 with a 95% confidence interval of 0.702-0.894, shown in Figure 4(a). The overall AUC value indicates the classification performance for this sample of data, which included tumors from a wide size range of 0.8 to 8 mm in diameter. The optimal cutoff, with sensitivity and specificity weighed equally, resulted in a specificity of 0.86 and a sensitivity of 0.65, at a reader assessment rank of 3.

The overall performance of the study, represented by the ROC curve, is dependent on the distribution of tumor sizes included in the study. Therefore, in order to characterize the relationship between reader performance and tumor diameter, we applied ROC regression, as described in equation (2). Regression analysis indicated that tumor diameter had a significant effect on the ROC curve, with *p* = 0.018 and a coefficient of 0.343.  Figure 4(b) shows the expected ROC curves for 3 different tumor sizes, chosen from the quartiles of the tumor diameters: 1.37, 2.32, and 5.09 mm. The area under the ROC curve and 95% confidence intervals for these tumor diameters are 0.670 (0.593, 0.766), 0.777 (0.723, 0.860), and 0.956 (0.907, 0.997), also listed in Table 1.

**Figure 5:**
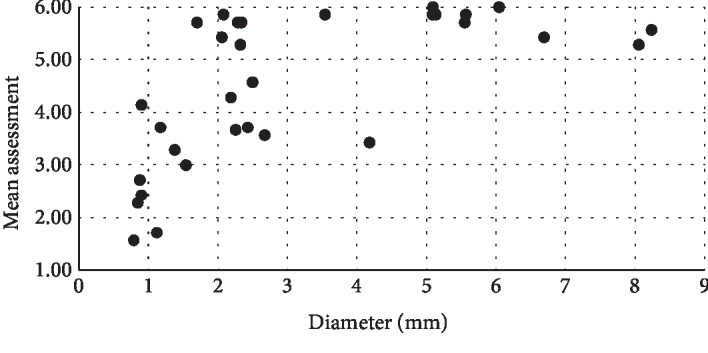
Average assessment from 7 readers vs. tumor size. Tumors larger than 3 mm received high ratings, indicating strong confidence of tumor presence.

The average rating for a control image was 2.2 ± 0.5, with most control images consistently rated between 1 and 3, while the mean rating for tumors among all readers was 4.5 ± 1.4, with ratings for tumors being noticeably more variable across tumor diameters.  Figure 5 shows the mean reader assessment rank for the 31 tumors versus tumor diameter. Scores for the 21 tumors smaller than 3 mm in diameter received a wide range of scores between 1.57 and 5.86, with a mean and standard deviation of 3.92 ± 1.35. However, the 10 tumors larger than 3 mm in diameter received consistently higher scores with a mean and standard deviation of 5.53 ± 0.7. These results suggest that tumor size strongly influenced the ability of readers to identify the vascular geometry tumors.

Overall, the specificity of the reader study was better than the sensitivity, meaning that false positives are uncommon using the qualitative reader study approach for classification. Different clinical applications may require different levels of sensitivity or specificity to provide useful classification.  Table 2 lists the sensitivity and specificity values that result from requiring either a sensitivity or a specificity level of 0.9. For 1.37 and 2.3 mm tumors, sensitivity and specificity are expected to be suboptimal when the other metric is required to be 0.9, but for tumors approximately 5 mm in diameter, both the sensitivity and specificity are greater than 0.9.

### 3.2. Tortuosity Analysis and Clustering

Quantitative analysis of tortuosity included calculations of the distance metric and sum of angles metric for all vessels segmented in each image. Each image was summarized using mean tortuosity metrics in order to compare tumor and control images using the Wilcoxon rank sum. The mean and standard deviation of the distance metric was 8.89 ± 22.24 in tumors and 2.20 ± 2.72 in controls, with no significant difference between tumor images and controls (*p* = 0.11). The mean and standard deviation of the sum of angles metric was 3.61 ± 0.38 rad/mm for tumors and 3.09 ± 0.57 rad/mm for controls, resulting in a significant difference between the two groups, with *p* = 2.8 × 10^−3^.

The mean tortuosity metric can be used to classify an image by setting a simple threshold to determine if the image is more likely to belong to the tumor or the control group. ROC analysis was used to determine the efficacy of classification using mean tortuosity across the range of possible thresholds. The AUC for the distance metric was 0.682, and 0.824 for the sum of angles metric and listed in Table 3. These results are similar to the overall AUC found using the qualitative reader study approach (0.778). These results support the hypothesis that tortuosity is higher in tumors than controls and therefore can be used to determine if an individual image contains a tumor, whether using visual or quantitative methods of discrimination.

However, classification based on mean tortuosity diminishes the likelihood of detecting small tumors due to use of the mean value to represent the population of vessels within an entire image. Additionally, the use of summary statistics neglects the predictive value of heterogeneity and spatial information provided in the vascular images. Therefore, a clustering approach known as Density-Based Spatial Clustering in Applications with Noise (DBSCAN) was used to produce a classifier that accounts for spatial heterogeneity.

**Figure 6:**
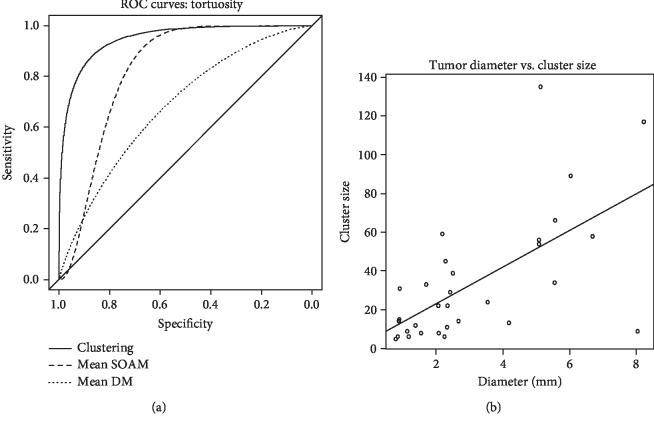
(a) ROC curves for differentiation between tumor and control images using tortuosity metrics. AUC = 0.95, 0.82, and 0.68. (b) Scatterplot of tumor diameter versus maximum cluster size and linear regression line (*p* = 1.3 × 10^−4^ and *R*^2^ = 0.37).

The vessels in each image were grouped into clusters using the DBSCAN algorithm, and maximum cluster size was chosen as the most predictive parameter for image classification. Other potential classification metrics generated by DBSCAN, such as total number of vessels clustered and number of clusters generated, had lower predictive value than the maximum cluster size. The mean and standard deviation of the maximum cluster size were much greater in tumor images (33.84 ± 32.76) than control images (5.41 ± 1.42). ROC analysis of maximum cluster size for image classification resulted in an area under the curve of 0.948 (95% CI: 0.829-0.977), as seen in Figure 6(a) and Table 3.

Weighing sensitivity and specificity equally, the optimal cutoff is a minimum cluster size of 8 vessels, resulting in a sensitivity of 0.871 and a specificity of 0.941, with 95% confidence intervals of 0.667-1.0 and 0.765-1.0, respectively. Maximum cluster size is positively correlated to tumor diameter, with a significant linear regression resulting in *p* = 1.3 × 10^−4^ and *R*^2^ = 0.37, as seen in Figure 6(b). Density-based spatial clustering of vessel-level tortuosity metrics provides the most accurate detection of tumors in these acoustic angiography images, with higher area under the ROC curve (0.95) than both image-level statistical analysis and reader study-based identification. The reader study approach resulted in an AUC of approximately 0.8, with high reader agreement (*W* = 0.778), and no significant differences between readers. These results are consistent with the trends presented by a previous study of 3D vascular morphology in liver tumors [36]. Though reader agreement was good, there was greater variation between the ratings of tumor images than those of controls, especially in small tumors. The tumors with the most variability in assessment level tended to have sparser vascular patterns and features that were visible in some of the 2D image frames but obscured in the maximum intensity projections. This suggests that images with vascular abnormalities visible in the maximum intensity projection resulted in simpler and more consistent ratings as tumors.

**Figure 7:**
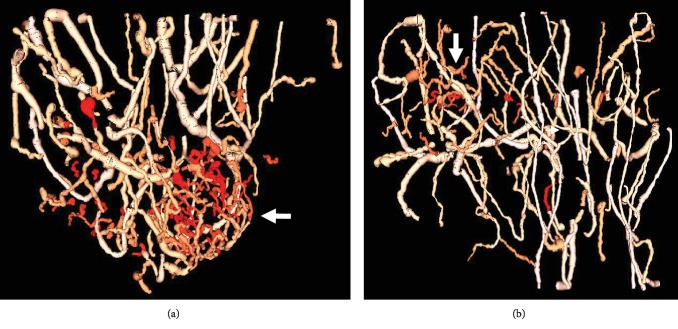
Renderings of segmented vasculature of tumors with color intensity showing tortuosity (SOAM). (a) shows a large tumor (5.1 mm) and (b) shows a smaller tumor (3.5 mm) with a cluster of tortuous vessels in the tumor region indicated by an arrow.

Three-dimensional tortuosity in small vessels can be difficult to identify in maximum intensity projections (such as those seen in Figure 2), due to multiple vessels overlapping each other in the projected volume, which can obscure the morphology of individual vessels. Therefore, image segmentation and quantitative analysis were also performed to calculate vascular tortuosity, which can be displayed as 3D objects instead of image projections, as seen in Figure 7. Two metrics of tortuosity, the sum of angles metric and distance metric, were computed, and the SOAM revealed a significant difference between tumor and control images while the DM did not. However, mean values do not consider spatial information, biasing the detection toward larger tumors. Therefore, the most reliable classification method was using the Density-Based Spatial Clustering in Applications with Noise (DBSCAN) to generate clusters using vessel-level tortuosity data and using the size of the cluster as a prediction metric. In this study of 48 images, tortuosity clustering-based classification resulted in an excellent area under the curve of 0.95.

This study demonstrates the performance of acoustic angiography for distinguishing tumors from healthy control tissue through a reader study approach and using quantitative analysis of vascular geometry. Both methods resulted in AUCs of 0.8 or better, but classification using clustering of quantitative tortuosity metrics yielded the highest AUC of 0.95. Unsurprisingly, both methods showed that the sensitivity of detection depended on the tumor diameter, with larger tumors detected with more accuracy. However, performance was still notable for tumors on the order of 5 mm, which would be considered very small in clinical diagnostics.

These results support the potential diagnostic benefit of microvascular imaging techniques in improving sensitivity and specificity over grayscale ultrasound. In addition to acoustic angiography, the technique of ultrasound localization microscopy (ULM), also referred to as super resolution (ultrasound) imaging, can provide even higher resolution microvascular images than described here [19, 44, 45] and can be combined with acoustic angiography in order to reduce the ULM preprocessing to separate microbubbles from the tissue clutter [46]. Although ULM still faces challenges with long acquisition and processing times for 3D data sets, it has the potential to resolve microvasculature smaller than 20 microns. Similarly, we hypothesize that photoacoustic imaging, which has similar capability to image the microvasculature in vivo [47], could also be used as a tool to identify the presence of malignant angiogenesis. A discussion of acoustic angiography relative to photoacoustic imaging has been provided previously by Gessner et al. [11].

## 4. Conclusions

An imaging technique with high resolution and high contrast to display vascular morphology has the potential to improve tumor diagnosis through the identification of the vascular features unique to tumor angiogenesis. Acoustic angiography, a contrast-enhanced ultrasound image technique, uses microbubble superharmonics to selectively image microvasculature as small as 100-200 *μ*m. These images can be used to identify images of tumors in a mouse model of breast cancer. Sensitivity was correlated to tumor diameter using visual assessment and quantitative approaches. The best discrimination was generated using a statistical clustering algorithm to identify clusters of tortuous vessels in images. This method resulted in an excellent area under the curve of 0.95 in a sample of 48 images with tumors ranging from 0.8 to 8 mm in diameter.

## Figures and Tables

**Table 1:** Reader study ROC area under the curve values.

Quartile	Diameter	AUC	Confidence interval^a^
1	1.37 mm	0.670	0.593-0.766
2 (median)	2.32 mm	0.777	0.723-0.860
3	5.09 mm	0.956	0.907-0.997
N/A	Combined	0.798	0.702-0.894

^a^95% confidence interval.

**Table 2:** Reader study sensitivity and specificity values.

Diameter	Sensitivity/specificity at 90% specificity	Sensitivity/specificity at 90% sensitivity
1.37 mm	0.461/0.9	0.9/0.239
2.32 mm	0.601/0.9	0.9/0.425
5.09 mm	0.902/0.9	0.9/0.908

**Table 3:** Tortuosity and clustering ROC area under the curve values.

Metric	AUC	Confidence interval^a^
Clustering	0.948	0.829-0.977
Mean SOAM	0.824	0.675-0.947
Mean DM	0.682	0.507-0.825
		

^a^95% confidence interval.

## Data Availability

The ultrasound image data used to support the findings of this study are available from the corresponding author upon request.
